# Pre-clinical development of BCG.HIVA(CAT) strain, an antibiotic-free selection strain for HIV-TB pediatric vaccine

**DOI:** 10.1186/1742-4690-9-S2-P352

**Published:** 2012-09-13

**Authors:** N Saubi, E Gea-Mallorquí, A Mbewe-Mvula, C Hurtado, J Gatell, T Hanke, J Joseph

**Affiliations:** 1 AIDS Research Unit, Hospital Clinic/IDIBAPS-HIVACAT, Barcelona, Spain; 2 The Jenner Institute, University of Oxford, Oxford, UK

## Background

Our starting platform was based on a heterologous BCG prime and MVA boost regimen delivering a common immunogen called HIVA. In this study, we have i) developed a BCG.HIVA^CAT^ strain containing an antibiotic free selection system (Cobra); ii) evaluated the specific HIV-1 immune responses induced after newborn BALB/c mice immunization with BCG.HIVA^CAT^ prime and MVA.HIVA.85A boost; iii) evaluated the specific-TB immune responses induced after newborn BALB/c mice immunization with BCG.HIVA^CAT^ prime and MVA.HIVA.85A boost and iv) evaluated the influence of age on specific HIV-1 immune responses using the same vaccination schedule.

## Methods

7-days-old newborn and 7-weeks-old adult mice were either left unvaccinated or vaccinated subcutaneously with 10^5^ cfu of BCG.HIVA^CAT^ or BCGwt, and 16 weeks later were boosted intramuscularly with 10^6^ pfu MVA.HIVA.85A. The mice were sacrificed 2 weeks later. The HIV-1 and TB-specific cellular immune responses were analyzed in spleen cells by intracellular cytokine staining and IFN-γ ELISPOT.

## Results

The frequencies of TB-specific CD8 ^+^ T-cells producing IFN-γ (P11 stimulation), and spleen cells producing IFN-γ (P11, P15 and PPD stimulation), were higher in BCG.HIVA^CAT^ or BCGwt primed and MVA.HIVA.85A boosted mice compared with mice vaccinated with MVA.HIVA.85A alone (i.e. 231, 108 and 24 sfu/10^6^ PPD stimulated splenocytes respectively). The specific HIV-1 immune responses (P18I10 stimulation) were lower in BCG.HIVA^CAT^ or BCGwt primed and MVA.HIVA.85A boosted mice compared with mice vaccinated with MVA.HIVA.85A alone (i.e. 270, 276 and 412 sfu/10^6^ P18I10 stimulated splenocytes respectively). When adult and newborn mice were immunized using the same vaccination schedule, the HIV-1-specific immune responses in adult mice were higher than in newborn mice (0.45% vs 0.2% CD8^+^ T-cells producing IFN γ).

## Conclusion

In conclusion we demonstrated the immunogenicity of BCG.HIVA^CAT^ and MVA.HIVA.85A in newborn mice but additional experiments should be performed in newborn mice testing different routes and doses that might provide different levels of immunogenicity.

